# The underused hip in ipsilaterally orthotics-dependent children

**DOI:** 10.1007/s11832-015-0667-7

**Published:** 2015-07-04

**Authors:** Asser Sallam, Christian M. Ziegler, Volkmar Jansson, Bernhard Heimkes

**Affiliations:** Department of Orthopedic Surgery, Physical Medicine and Rehabilitation, University Hospital of Munich (LMU), Campus Grosshadern, Marchioninistr. 15, 81377 Munich, Germany

**Keywords:** Orthotics/prosthetics-dependent children, Diseased hips, Healthy hips, Unloading coxa valga, Instrumental gait analysis, Mechanobiology of the hip

## Abstract

**Background:**

The aim of this investigation is the development of primarily healthy hips in children who have required orthoses/protheses over the long term due to ipsilateral distally located deformities of the leg. These children show ipsilateral in-toeing gait and Duchenne’s limping followed by a coxa valga antetorta and facultative hip decentration. A practical question is whether these hips are in danger of decompensation. An additional theoretical question is how the external shape and internal architecture changes if a primarily healthy hip is underused.

**Methods:**

Ten children with healthy hips who are unilaterally long-term orthotics/prosthetics-dependent agreed to undergo an instrumental gait analysis. The results were analyzed and correlated with clinical findings, a common activity score and planimetric radiographic data.

**Results:**

The intra-individual comparison revealed a number of significant changes in the hip of the deformed leg (*p* < 0.05). Clinically, the internal rotation was increased (15° ± 4.2°), while the external rotation was diminished (13° ± 1.3°). Radiologically, the projected caput–collum–diaphyseal angle, the lesser trochanter to articular surface distance and the head–shaft angle were increased by 11.1° ± 15.4°, 5.8 ± 4.2 mm and 11.9° ± 0.6°, respectively. Both the Sharp and acetabular angles were increased, the former by 3.6° ± 0.6° and the latter by 3.2° ± 0.6°. Kinetic gait analysis showed increased stride length (6.8 ± 3.7 cm), shortened stance phase (6.6 ± 1.6 %) and reduced forces transmitted to the ground (92.2 ± 34.3 N). The kinematic analysis showed increased hip abduction (14.0° ± 8.2°), while the pelvic obliquity was not significantly changed (0.01° ± 0.01°).

**Conclusions:**

Duchenne’s limp and lack of weight-bearing stress are the decisive pathogenic factors of the underused coxa valga and acetabular dysplasia. These changes follow the mechanobiological concept of “function modifies design”, which means function influences external shape and internal architecture of bones and joints. As a practical consequence we recommend that one pelvic radiograph be performed as a precaution at the end of puberty of children with these conditions.

**Level of evidence:**

Level II retrospective study.

**Electronic supplementary material:**

The online version of this article (doi:10.1007/s11832-015-0667-7) contains supplementary material, which is available to authorized users.

## Introduction

**Fig. 1:**
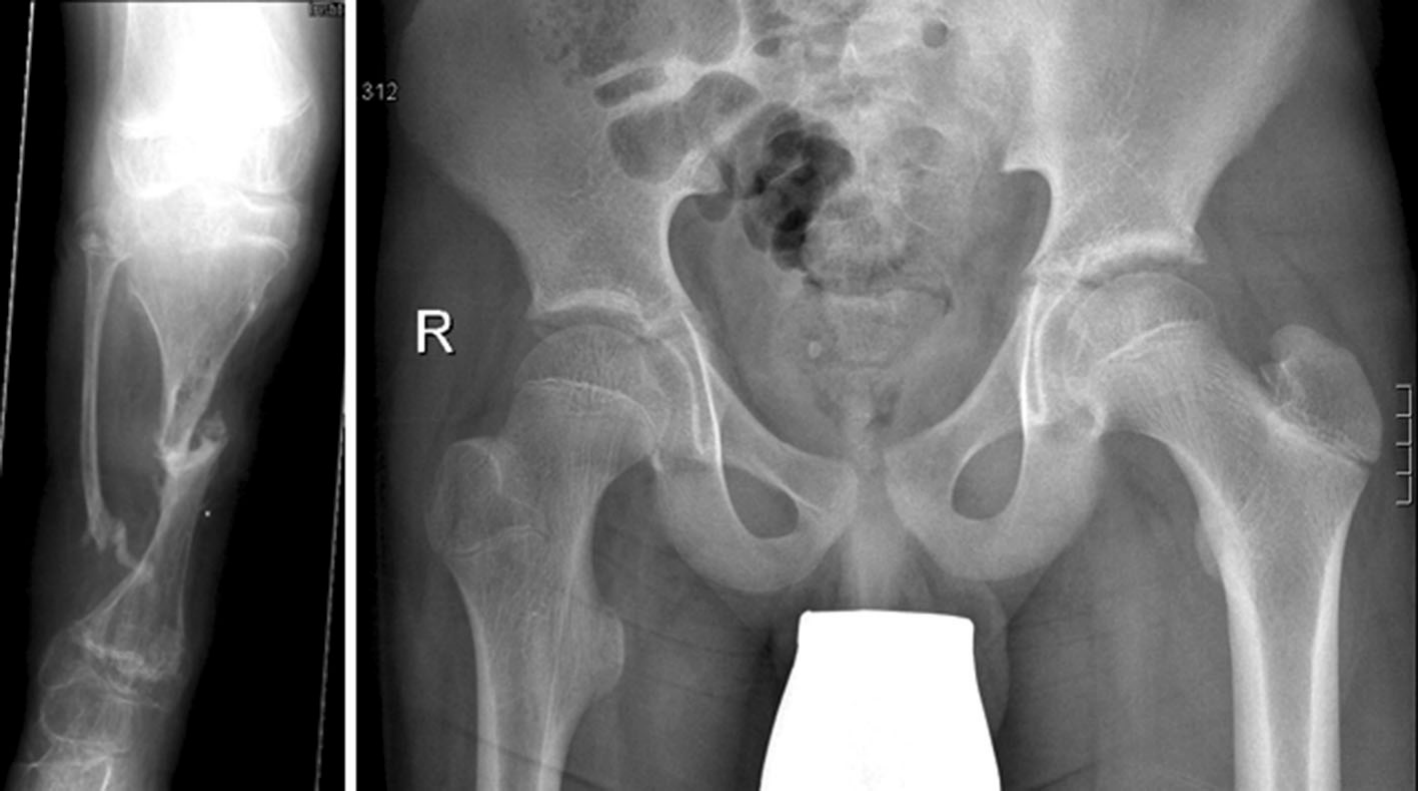
Unilateral coxa valga and slight acetabular dysplasia of the right side in a 12-year-old boy who had been orthotic dependent for 11 years due to a right-side congenital tibial pseudoarthrosis. Ullmann–Sharp angle: right side 44°, left side 40°. Neck–shaft angle: right side 156.8°, left side 136.1°. Head–shaft angle: right side 76°, left side 63°

A healthy child burdens the lower limb joints daily about 10,000–15,000 times during walking and running [1–4]. Based on clinical experience we have noted that children with primarily healthy hips but who have been orthotics/prosthetics-dependent for many years due to underlying limb length discrepancy (LLD), below-knee amputation and deformities at the knee, leg or foot, develop coxa valga (Fig. 1).

Studies using several biomechanical models of the adult [5–8] and infantile hip [9, 10] have revealed that the forces acting on the hip joint are determined firstly by the external moment due to the body weight and by the counteracting moment of the hip abductors and secondly by the forces acting on the greater trochanter, which are the hip abductors, parts of the knee extensors and the dynamic input of the iliotibial band. These forces may be reduced either by reduction of the external forces, such as while using orthotics or prosthetics, or by shortening the body weight lever arm by Duchenne’s limp [11].

Accordingly, in children who have been orthotics/prosthetics-dependent over the long term, the weight-bearing stress is reduced, which results in the growth plate of the femoral head acquiring a horizontal alignment due to changes in both the direction and amount of muscle forces acting on the hip joint. This in turn results in an increased femoral neck–shaft angle and, consequently, the development of coxa valga.

A theoretical purpose of this study was to clarify, by means of instrumental gait analysis, the gait criteria of children with primarily healthy hips but who suffer from acquired or congenital leg deformities and have become permanently orthotics/prosthetics-dependent, in order to elucidate the possible pathogenic factors that might lead to development of an unloading coxa valga and secondary hip dysplasia. An additional practical purpose was to analyze whether the hips of these children are in danger of decompensation.

## Materials and methods

### Clinical and demographic data

This study is a retrospective review of all orthotics/prosthetics-dependent children of both genders who suffered unilateral congenital or acquired deformities distal to the knee but with primarily ipsilateral healthy hips between 1996 and 2013. Children younger than 6 years or those with neuromuscular or metabolic diseases were excluded from the study. The contralateral healthy leg was used for comparison. Prior to initiation of the study, approval was granted by the Institutional Review Board and the Research Ethics Committee of University Hospital of Munich.

A total of 12 children (9 males, 3 females) were eligible for study inclusion based on the initial data trawl of the electronic patient database and underwent clinical and radiological hip examination. Among these, two children refused to undergo the instrumental gait analysis. Thus, the final study cohort comprised ten children [8 (80 %) males, 2 females (20 %)].

The deformities were etiologically based on five entities: local neurofibromatosis or tibial pseudoarthrosis (*n* = 5), severe congenital club foot (*n* = 1), congenital tibial longitudinal defects (*n* = 2), congenital fibular longitudinal defects (*n* = 3) and tibial deformation following Ewing’s sarcoma (*n* = 1). The mean age of the patients was 12.56 (range 6–18.5) years, and the average unloading period was 10.6 (range 3.25–16.33) years. Seven children had a diseased left leg and three had an affected right side.

The clinical parameters assessed were LLD, which was determined by measuring the distance (in centimeters) of the anterior superior iliac spine to the medial malleolus, goniometric measurement of hip range of motion (ROM), which was determined according to the Neutral-0-Method [12], Duchenne’s limp when the upper part of the body could not be stabilized during gait, resulting in a rocking motion towards the diseased side and UCLA score [13, 14].

### Radiological measurements

We analyzed a total of 17 pelvic radiographs. Only technically accepted images were included. The parameters assessed were: the projected angle between the femoral neck and diaphysis (pCCD) as described by Müller [15] and Hefti [16], the lesser trochanter to articular surface distance (LTA) as described by McCarthy and Weiner [17], the angle between the femoral epiphyseal line and diaphysis (CF) as described by Birkenmaier [18], the Sharp–Ullmann angle [19] and Hilgenreiner’s acetabular (AC) angle [20]. The pCCD and CF angles as well as the LTA distance reflect the growing femoral head morphology, biomechanics and the reactions of the epiphyseal plates to the biomechanical changes. Similarly, both the Sharp–Ullmann and AC angles function as indicators of acetabular morphology and biomechanics.

### Instrumental gait analysis

Kinematic and kinetic data as well as the time–distance parameter were acquired and analyzed to describe fundamental gait characteristics of our children. Gait analysis was performed in the three dimensions using the Zebris Treadmill System (FDM-T System with the CMS-HS System; Zebris Medical GmbH, Isny in Allgäu, Germany). The following anatomical landmarks were tracked by applying a marker to the left midfoot, left distal thigh, right midfoot, right distal thigh and the sacrum. The ground plane was then calibrated using a pointer marker, followed by determination of the following anatomical points: sacrum, anterior superior iliac spine (right/left), knee external joint line (right/left), knee internal joint line (right/left), ankle lateral malleolus (right/left), ankle medial malleolus (right/left), lower heel (right/left) and tip of big toe (right/left).

The children were asked to walk on the treadmill at a self-selected speed and the following parameters were collected: time–distance parameter (speed, step length and cadence), phases of the gait cycle, kinetic data (forces transmitted to and pressure on the ground) and kinematic data (pelvic tilt, rotation and obliquity as well as hip flexion/extension, abduction/adduction and internal/external rotation).

## Results

### Clinical results

The clinical results are presented in Table 1. The mean UCLA score was 5.83 (range 2–10) and the LLD was 8.8 (range 1–32) cm on the diseased side. On the diseased side the hip internal rotation was significantly increased (15° ± 4.2°) and the external rotation significantly diminished (13° ± 1.3°) (*p* < 0.05).

**Table 1:** Measurements of limb length discrepancy (LLD), lower limb length and hip range of motion on both the diseased and healthy sides of the patients

Measurements	Diseased side	Healthy side	*t* test	*p* value
LLD (in cm) (in standing position without orthosis)	8.8 ± 9.54	–	–	–
Lower limb length (in cm) (lying)	70.35 ± 13.98	79.15 ± 13.59	2.92	<0.05
Thigh length	44.65 ± 6.53	45.4 ± 5.96	0.87	>0.05
Leg length	26.1 ± 11.5	34.15 ± 7.27	2.33	<0.05
Hip joint				
Extension	17 ± 4.83	17 ± 4.83	–	–
Flexion	138.5 ± 9.14	142 ± 7.89	1.76	>0.05
Abduction	49 ± 13.5	49 ± 13.08	0.00	>0.05
Adduction	38 ± 8.1	37 ± 7.89	1.00	>0.05
External rotation (in extension)	22.5 ± 20.45	35 ± 21.6	3.049	<0.05
Internal rotation (in extension)	66.5 ± 11.8	53.5 ± 14.15	5.46	<0.001
External rotation (in flexion)	22.5 ± 20.45	35.5 ± 21.79	3.07	<0.05
Internal rotation (in flexion)	66.5 ± 11.8	51.5 ± 16.58	4.61	<0.01

*p* < 0.05 indicates significant difference

Values are presented as the mean ± standard deviation (SD)

*LLD* limb length discrepancy (LLD)

### Radiological results

**Fig. 2:**
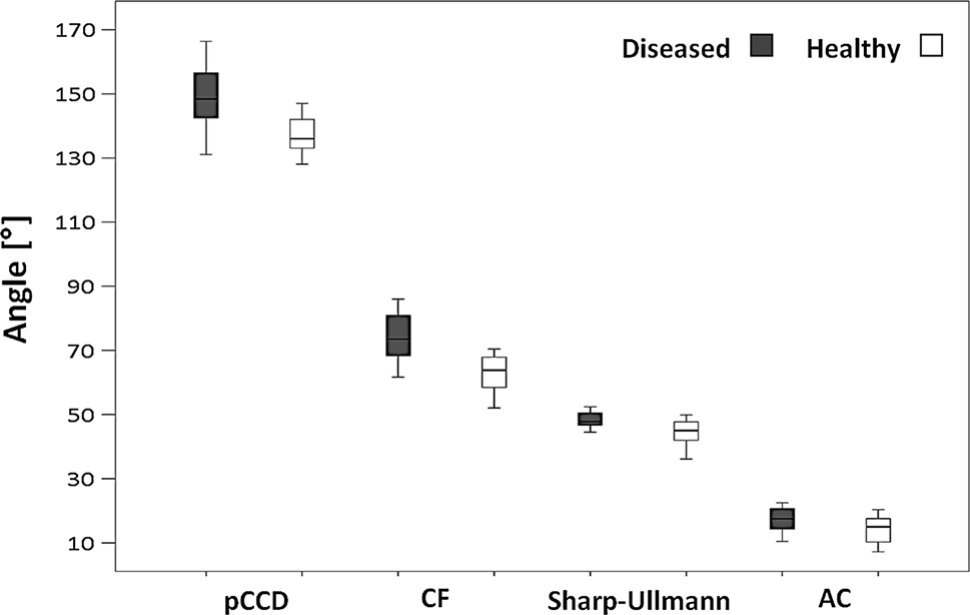
Femoral and acetabular geometry.* Boxplot* showing the projected angle between the femoral neck and diaphysis (caput collum diaphysis angle, *pCCD*), head–shaft angle (*CF*), Sharp–Ullmann angle and acetabular (*AC*) angle on the diseased and healthy sides. Four patients showed slight acetabular dysplasias according to Tönnis [21].

The intra-individual comparison between both sides revealed a number of changes in the hip of the deformed leg:

Regarding the changes in the proximal femoral end, the pCCD angle (Fig. 2) was age dependent, with a significantly higher mean value on the diseased side (*p* < 0.001). When compared to the normal age- and gender-specific reference values [12], the observed values indicate a tendency to develop coxa valga (148.05° ± 11.04°) on the diseased side. The mean LTA was also significantly increased (*p* < 0.01) compared with the healthy side (8.07 ± 2.14 and 7.49 ± 1.72 cm, respectively). Additionally, the CF angle (Fig. 2), which indicates the horizontal orientation of the epiphyseal plate, was significantly higher (*p* < 0.001) on the diseased side (74.86° ± 8.79°) than on the healthy one (62.93° ± 8.19°).

Both the Sharp-Ullmann and AC angles (Fig. 2) were significantly higher (*p* < 0.01) on the diseased side (45.85° ± 4.97° and 17.29° ± 3.8°, respectively) than on the healthy one (42.29° ± 5.6° and 14.04° ± 4.43°, respectively), indicating shallower acetabulum with increased inclination. Four patients showed slight acetabular dysplasias according to the grading of Tönnis [21]. The horizontal line in the box represents the statistical median, the top and bottom margins of the box represent the 25th and the 75th percentile respectively. The whiskers mark the 5th and 95th percentile.

### Gait analysis results

The results of the gait analysis are shown in Table 2. Analysis of the time–distance parameter of our patient series revealed a mean cadence of 82 ± 25.02 steps/min and a mean speed of 1.39 ± 0.64 km/h. Surprisingly, we observed a significantly longer step length and time (*p* < 0.05) on the diseased side (34.29 ± 13.59 cm and 1.25 ± 0.82 s, respectively) than on the healthy one (27.5 ± 9.89 cm and 0.8 ± 0.3 s, respectively). We also found a significantly shorter stance phase (66.77 ± 5.92 %) on the diseased side (*p* < 0.05) than on the healthy one (73.35 ± 7.5 %).

**Table 2:** Comparison of parameters of instrumental gait analysis on both the diseased and healthy sides of the patients

Parameters of the instrumental gait analysis	Diseased side	Healthy side	*t* test	*p* value
Step length (cm)	34.2.99 ± 13.59	27.5 ± 9.89		<0.05
Step time (s)	1.25 ± 0.82	0.8 ± 0.3		<0.05
Gait cycle				
Stance phase (%)	66.77 ± 5.92	73.35 ± 7.5		<0.01
Initial double support (%)	23.02 ± 9.06	20.16 ± 7.62		>0.05
Midstance (%)	25.32 ± 8.51	31.96 ± 7.73		<0.01
Terminal stance (%)	18.6 ± 6.4	21.21 ± 6.79		>0.05
Swing phase (%)	33.22 ± 5.94	26.65 ± 7.5		<0.01
Kinetic gait analysis				
Forces transmitted to the ground (N)	295.55 ± 124.54	387.77 ± 158.78		<0.05
Pressure on the ground (N/cm^2^)	12.63 ± 2.42	22.52 ± 15.59		<0.05
Kinematic gait analysis				
Pelvic obliquity (°)	1.25 ± 10.27	1.26 ± 10.28	−0.7	>0.05
Hip adduction (°)	–	11.78 ± 7.47		–
Hip abduction (°)	13.99 ± 8.23	–		–
Hip internal rotation (°)	13.39 ± 12.59	16.21 ± 26.18		>0.05
Hip external rotation (°)	7.66 ± 13.7	6.47 ± 8.5		>0.05

*p* < 0.05 indicates significant difference

Values are presented as the mean ± SD

The lower limb length discrepancies were corrected by the orthoses to ≤1 cm

**Fig. 3:**
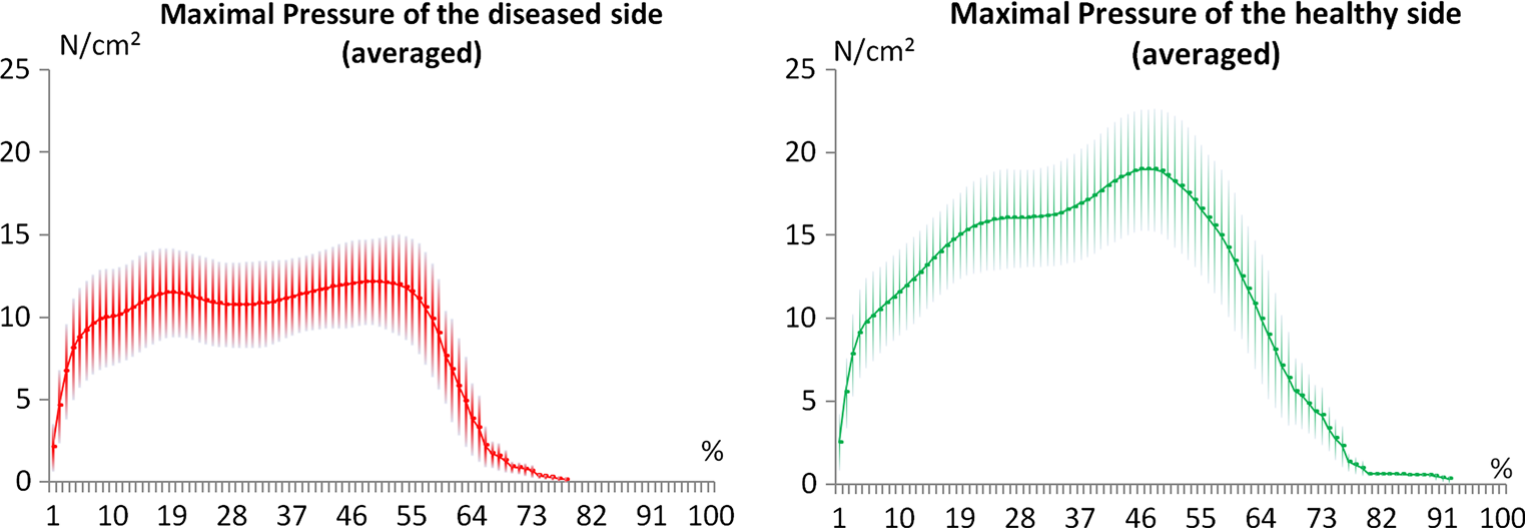
Kinetic gait analysis. Pressure on the ground of the diseased versus the healthy side (N/cm^2^)

Kinetic gait analysis revealed that compared to the healthy limb, significantly lower forces were transmitted from the diseased limb to the ground (295.55 ± 124.54 N) with significantly lower pressure (12.63 ± 2.42 N/cm^2^) on the treadmill (*p* < 0.05) (Fig 3).

**Fig. 4:**
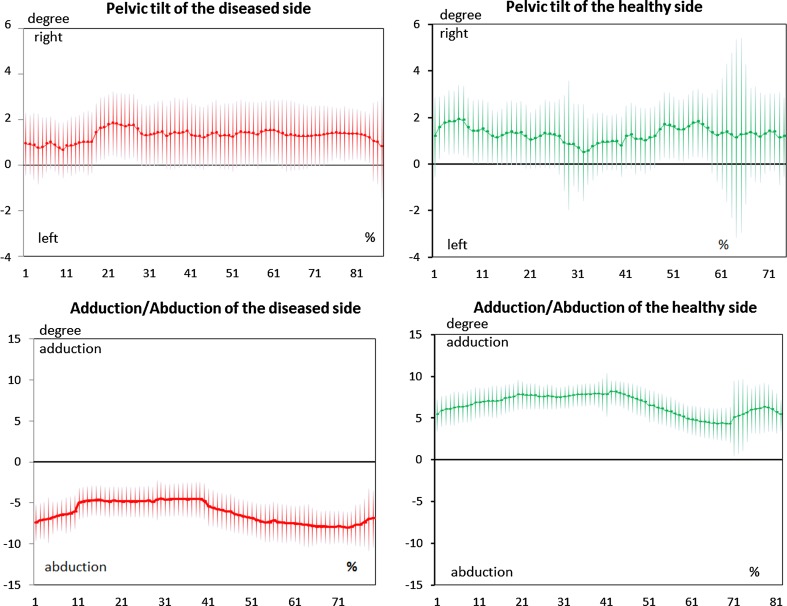
Kinematic gait analysis. Pelvic tilt and position of the leg in the coronal plane of the diseased versus healthy side

Kinematic analysis revealed that the hip of the diseased limb in the sagittal plane was exclusively abducted during walking while that of the healthy limb was only adducted. The pelvic obliquity was nearly similar on both sides (*p* > 0.05) (Fig 4).

## Discussion

### Discussion of materials and methods

There were a number of limitations to our study. First, the number of cases was very small due to the rarity of these deformities in children. Second, a comparative control group of normal healthy children was not available. However, we used clinical and radiological assessments as well as gait analysis in the review of patients’ data in order to make a precise evaluation of the diseased side versus the other healthy side. In addition, inter-observer bias of pelvic x-ray measurements per examiner was limited by determining the mean of three measurements of each radiological angle or distance. Additionally, to improve the intra- and inter-individual reliability, two examiners measured the hip ROM. Finally, a calibration of the gait analysis system was performed before each measurement in order to avoid the systemic error.

Another limitation was the retrospective character of our radiological measurements. As a consequence of this study design, the geometrical analysis of the hips was based on planimetrical radiography and is therefore inferior data obtained from three-dimensional clinical and anthropological studies [22–25]. We present the projected and not the anatomical neck–shaft angle. The reduced external and enlarged internal rotation of all diseased hips, however, suggests that all patients suffered from a coxa valga antetorta.

### Discussion of results

Our results confirm the development of an underused or “unloading” coxa valga [26] and secondary acetabular dysplasia in unilaterally orthotics/prosthetics-dependent children. The increased radiological measures of the proximal femoral end (pCCD, CF angles and LTA) obtained in this study support the findings of previous (English language) [27, 28] and very early (German) studies [29, 30] where the authors observed a secondary coxa valga in children with reduced weight-bearing due to deformities, leg length differences or amputations. In terms of acetabular morphology, a slight secondary dysplasia (as evidenced by increased Sharp–Ullmann and AC angles) developed, in accordance with findings reported by Mau [31].

**Fig. 5:**
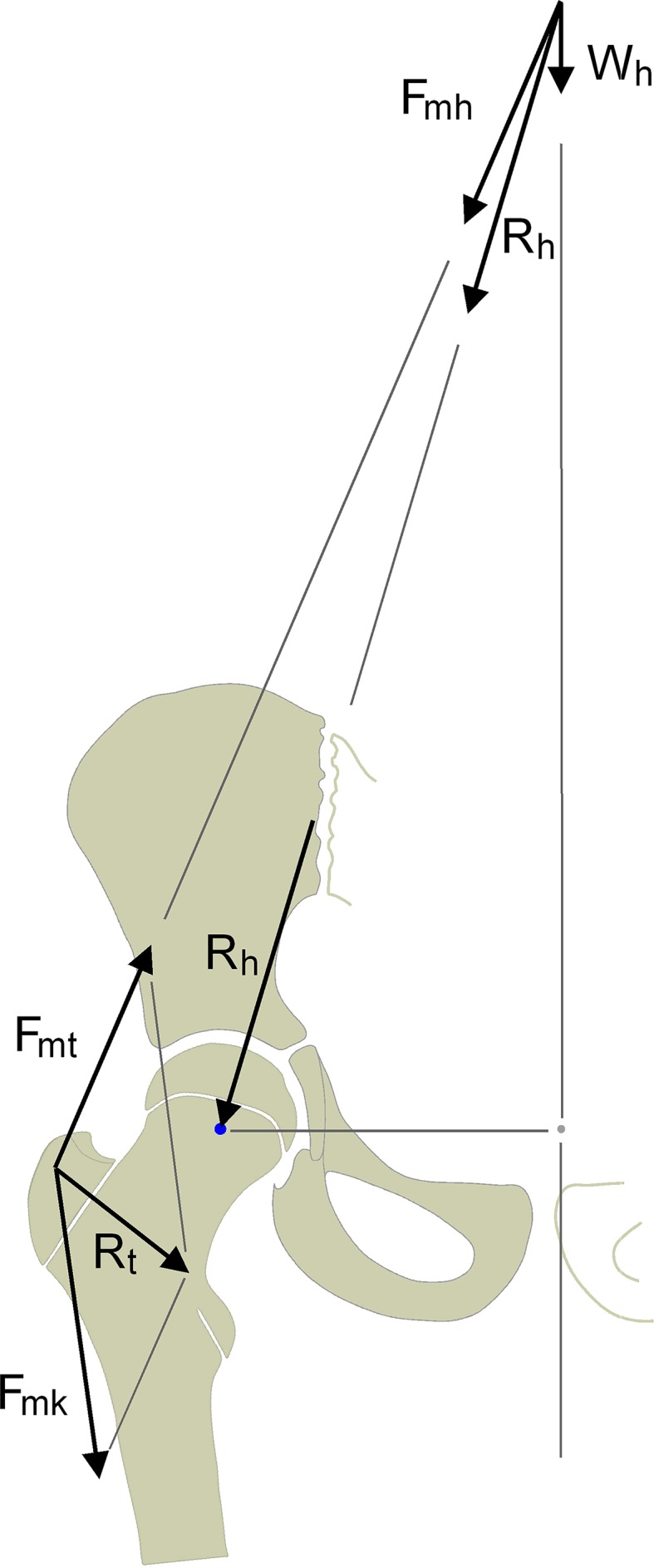
The proximal femur is stressed by two resulting forces, namely, the hip joint contact force (*R*_*h*_) and the resulting trochanteric force (*R*_*t*_). *R*_*t*_ is the vectorial sum of the resulting force exerted by all the abductor muscles (*F*_*mt*_) and the force composed of the traction on the iliotibial tract and the force exerted by the knee extensors (*F*_*mk*_) which are connected by the vastus lateralis muscle to the greater trochanter apophysis [8, 9]. *Wh* partial body weight acting on the hip during the stance phase of the gait

Familiarity with the mechanisms and forces that influence a normal infantile hip is needed to understand the natural history of the underused coxa valga. These mechanisms and forces can be explained by the so-called “clustered-pillar-concept” (Fig. 5), which was first published in 1993 [9] and again in 2009 [8] and applied to the normal growth of the infantile hip in 1997 [32]. According to similar concepts in biomechanical engineering [6] and mechanobiological studies [5, 7, 10, 33], and in contrast to the historical models of Koch [34] and Pauwels [11], this concept involves modification of the shape of the coxal femoral end by two resulting forces. The first of these is *R*_h_, the hip joint contact force, and the second one is the resulting force *R*_t_ which provides compressive stress to the greater trochanter growth plate from a cranio-lateral direction by a vasto-gluteal muscle sling and by the iliotibial tract.

A summary of the kinetic results of our gait analysis includes a longer step length and duration, a shortened stance phase and a lower ground reaction force on the diseased side. The lack of proprioception from the diseased limb and the weight of the prosthesis itself might compel the children to lengthen their step and take a longer time to move their limbs during a prolonged swing phase. Our results are in agreement with those of Pereira [35], who reported that patients with symptomatic leg-length discrepancies showed not only small force peaks during walking but also needed a longer time to reach these peaks.

**Fig. 6:**
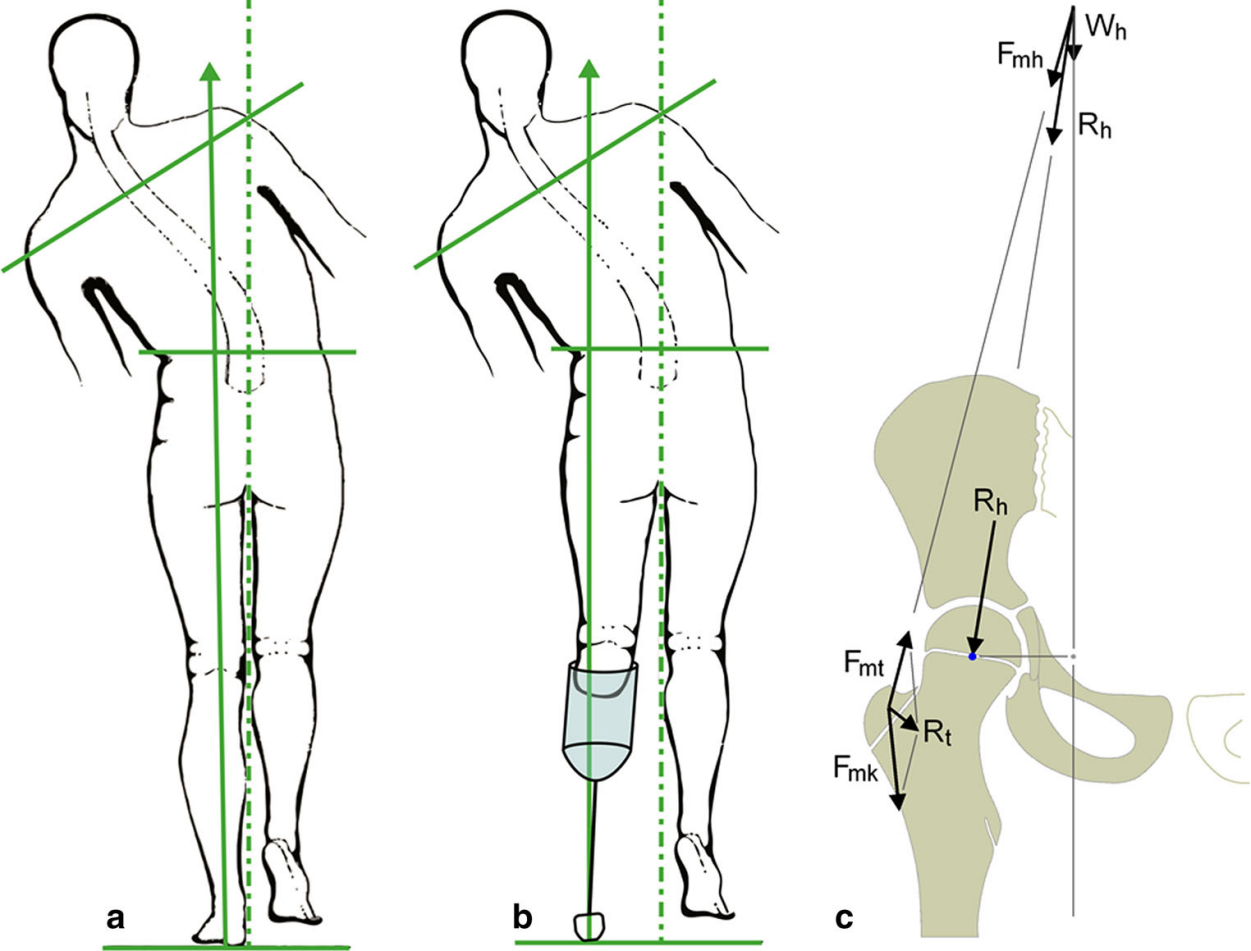
Schematic drawing of the gait of orthotics-dependent children against to a conventional Duchenne limp. *Broken line* Midline, *solid line* center of gravity line. **a** Conventional Duchenne limp with narrow walking base according to Schröter et al. [26], **b** typical Duchenne limp of the orthotic-dependent child with abducted leg, **c** Duchenne limp shortens the body lever arm of the hip and reduces the forces of the hip abductors. The load on the greater trochanter growth plate is diminished, this mechanism leads to a coxa valga

For our comparison, we assumed that in normal walking the center of body mass is placed over the weight-bearing leg by a lateral displacement in the stance phase [36]. This is achieved by a slight adduction of the leg and a lateral shift of the trunk (“physiologic Duchenne”); simultaneously, there is a pelvic tilt that reduces the vertical displacement of the center of mass (“physiologic Trendelenburg”) and acts as a shock absorber [37]. In contrast, we found that all of our patients abducted the leg and widened the step towards the diseased side. Walking with an abducted leg without any further compensation is not possible, as the center of mass would not be positioned vertically to the weight-bearing foot. Consequently, the abducted leg compels the patients to use the second possible mechanism of shifting the center of gravity laterally: according to the schematic drawing in Fig. 6b they have to shift the trunk to the affected side.

Another surprising result is that the pelvic tilt of the diseased side does not decrease as normally expected in the stance phase. One possible explanation is that patients use their affected leg more cautiously with longer strides and at less maximum pressure, as seen in our results. This adaption may reduce the dynamic forces which are one cause of the decreased physiologic pelvic tilt.

According to the computations of Pauwels [11], Duchenne limping shortens the body lever arm and secondarily weakens the abductor muscles. This mechanism unfortunately reduces the growth rate of the greater trochanter growth plate and leads to a coxa valga. Duchenne limping therefore would appear to be the most important pathogenic factor in the development of an underused coxa valga. These bony changes follow the common biomechanical law “Function modifies Design” and confirm the theory of causal histogenesis in that the external shape and internal architecture of the hip joint reflect the loading history of the hip [38].

Based on our radiological data, four patients had only slight acetabular dysplasias according to the grading of Tönnis [21] without any need of operative correction. We conclude that long-term orthotic-dependent patients with an underused coxa valga are likely not in danger of developing severe acetabular dysplasias. As a practical consequence of our study, we recommend discontinuation of periodical X-ray controls of the hips, to be replaced by one pelvic radiograph at the end of puberty as a precautionary measure. If distally orthotic-dependent patients suffer from additional diseases that promote coxa valga and hip decentration, the possibility of orthosis as an additional risk factor for hip migration should be considered.

## Electronic supplementary material

Supplementary material 1 (MPEG 223039 kb)
